# Learning self-supervised molecular representations for drug–drug interaction prediction

**DOI:** 10.1186/s12859-024-05643-7

**Published:** 2024-01-30

**Authors:** Rogia Kpanou, Patrick Dallaire, Elsa Rousseau, Jacques Corbeil

**Affiliations:** 1 Département d’informatique et Génie Logiciel, Université Laval, Québec City, QC Canada; 2 Centre de Recherche en Données Massives de l’Université Laval, Québec City, QC Canada; 3 Centre de Recherche en Infectiologie de l’Université Laval, Axe Maladies Infectieuses et Immunitaires, Centre de Recherche du CHU de Québec-Université Laval, Québec City, QC Canada; 4 Département de Médecine Moléculaire, Faculté de Médecine, Université Laval, Québec City, QC Canada; 5 Centre Nutrition, Santé et Société (NUTRISS), Institute of Nutrition and Functional Foods (INAF), Université Laval, Québec City, QC Canada

**Keywords:** Drug–drug interactions, Contrastive learning, Deep neural networks, Representation learning, Fine-tuning, Smiles enumeration, Transfer learning

## Abstract

Drug–drug interactions (DDI) are a critical concern in healthcare due to their potential to cause adverse effects and compromise patient safety. Supervised machine learning models for DDI prediction need to be optimized to learn abstract, transferable features, and generalize to larger chemical spaces, primarily due to the scarcity of high-quality labeled DDI data. Inspired by recent advances in computer vision, we present SMR–DDI, a self-supervised framework that leverages contrastive learning to embed drugs into a scaffold-based feature space. Molecular scaffolds represent the core structural motifs that drive pharmacological activities, making them valuable for learning informative representations. Specifically, we pre-trained SMR–DDI on a large-scale unlabeled molecular dataset. We generated augmented views for each molecule via SMILES enumeration and optimized the embedding process through contrastive loss minimization between views. This enables the model to capture relevant and robust molecular features while reducing noise. We then transfer the learned representations for the downstream prediction of DDI. Experiments show that the new feature space has comparable expressivity to state-of-the-art molecular representations and achieved competitive DDI prediction results while training on less data. Additional investigations also revealed that pre-training on more extensive and diverse unlabeled molecular datasets improved the model’s capability to embed molecules more effectively. Our results highlight contrastive learning as a promising approach for DDI prediction that can identify potentially hazardous drug combinations using only structural information.

**Supplementary Information:**

The online version contains supplementary material available at 10.1186/s12859-024-05643-7.

## Introduction

Drug–drug interactions (DDIs) occur when one drug affects another drug's efficacy or therapeutic effects. Early detection of DDIs is critical to patient safety and quality of care. Identifying potential drug–drug interactions traditionally requires labor-intensive pair-by-pair experiments in vitro and in vivo.

In silico methods have recently become very popular for the prediction of DDIs. They provide a cost-effective and efficient screening tool for DDIs. However, representing molecules is a challenging task. Different molecular representations have been proposed, such as chemical descriptors and fingerprints [1]. Chemical descriptors are quantitative measurements of a molecule's structural, physical, or chemical properties. They are derived from molecular structures, including size, shape, and connectivity. They are usually calculated based on predefined rules or mathematical algorithms. Some descriptors are computationally intensive or time-consuming to calculate and are less suitable for large-scale applications. The most commonly used descriptors are the molecular weight and the logarithmic partition coefficient (logP). Fingerprints condense the structure of a molecule into a binary bit string. Each bit represents a specific atom, ring, or functional group. In this way, fixed-length fingerprints are generated, resulting in representations of equal length for all compounds. Although these representations are efficient, this compression can lead to a loss of information and result in similarity between longer, complex molecules and smaller, simpler molecules. Some fingerprints, such as MACCs keys or PubChem fingerprints (PFPs), are limited to fragments in the libraries on which they are built. Other fingerprints, such as ECFPS, focus only on local structural features, not global or long-range structural features that may be critical for some applications.

Many methods have investigated the predictive power of molecular descriptors and fingerprints for predicting DDI [2–4]. These methods mainly fall under the category of similarity-based approaches. They rely on the assessment of similarity between drugs to infer potential interactions. In similarity-based approaches, drugs are compared based on their structural and chemical characteristics. Pairwise similarity measures such as the Tanimoto coefficient or the Jaccard distance are calculated. Higher similarity values indicate a higher probability of interaction between drugs. Additional fingerprints were also used to characterize different aspects of drugs. These include side effect profiles [5], interaction profiles [6, 7], and target profiles [8–12]. Side effect profiles provide information about the observed adverse effects associated with drugs. Interaction profiles describe the partners involved in drug interactions and record the drugs with which a particular drug tends to interact. Finally, target profiles describe the biological targets with which drugs interact.

Deep learning-based methods have shown great potential to improve the accuracy of DDI prediction by learning more informative and discriminative features directly from the raw molecular structures. In contrast to similarity-based approaches, these methods aim to let the neural network discover the most valuable patterns for predicting DDI. The two most commonly used representations of these approaches are the Simplified Molecular Input Line Entry System (SMILES) and molecular graphs constructed from SMILES. SMILES serves as a textual encoding of molecular structures, providing a compact representation for analysis and interpretation. Most known SMILES-based models for the prediction of DDI are inspired by natural language processing (NLP) techniques and use layers of recurrent neural networks (RNNs), long short-term memory (LSTM), and convolutional neural networks (CNNs) [4, 11–13]. Graph-based models rely on graph convolutional networks (GCN) layers to process molecular graphs and capture key structural features and relationships [14, 15].

Despite the success of these methods, some limitations still need to be addressed. These methods rely on large amounts of labeled data, which can be expensive and time-consuming. In addition, they mainly focus on predicting interactions between known drugs. They have been shown to perform poorly on predicting interactions between new drugs that have not been previously observed [16–19].

In machine learning approaches, transfer learning (TL) is often used when only a limited amount of labeled data is available [20, 21]. Transfer learning has been successfully applied in many fields, e.g., text classification [13, 22], image classification [23], and more recently, drug discovery [24]. Transfer learning uses pre-trained models on large datasets. Instead of training a model from scratch on a small dataset, the pre-trained model serves as a starting point. This reduces the data and time required for training and improves downstream tasks. TL can be supervised, self-supervised, or unsupervised. In recent years, self-supervised TL has gained more popularity [25]. Self-supervised models are more robust than models trained in a supervised manner. They are trained to learn representations independent of the specific downstream task. The learned features are more general and abstract because they do not rely on task-specific labels during training. This can improve generalization when the trained model is transferred to other tasks or domains. Contrastive learning is a widely used self-supervised learning technique that focuses on maximizing the similarity between different augmented views of the same object and minimizing the similarity between views of different objects. Contrastive learning has been used primarily in computer vision tasks [25]. It has been successfully applied to various tasks, including image classification, object detection, semantic segmentation, and image generation. One of the main advantages of contrastive learning is that it does not require manual annotation of the data and thus can easily scale to large amounts of unlabeled data. Recent advances in contrastive learning methods, such as SimCLR [26] and SwAV [27], have achieved state-of-the-art performance on benchmark datasets and tasks. These results have sparked great interest in the use of contrastive learning in other domains, including natural language processing and reinforcement learning.

Here, we present a novel self-supervised molecular representation for DDI prediction, SMR-DDI. SMR-DDI uses contrastive learning to compare augmented views of canonical SMILES using SMILES enumeration. We pre-trained a 1D-CNN encoder-decoder-like architecture on a large unlabeled molecular dataset using contrastive loss to minimize the differences between canonical and randomized SMILES. We then fine-tuned the encoder on a smaller labeled DDI dataset. To evaluate the richness of our feature space, we compared it to various state-of-the-art molecular representations while simulating different real-life use cases to validate its robustness and generalization. We also performed several ablation experiments to evaluate the impact of pre-training on our DDI prediction. In addition, we investigated the impact of pre-training with a more diverse molecular dataset and comprehensively analyzed the DDI dataset to gain insights into its properties. These analyses help to understand the model's performance and improve the predictions.

Notably, our method showed performance comparable to or sometimes better than the state-of-the-art, confirming the effectiveness of pre-training with contrastive learning for DDI prediction. Using a contrastive approach, we learned rich molecular representations for drugs with comparable predictive power to state-of-the-art molecular representations. Pre-training with a larger dataset also helped the prediction model to generalize better. The experiments showed that our molecular representation is not fixed but benefits positively from the chemical diversity in the training dataset. This flexibility makes the proposed molecular representation particularly valuable in real-world scenarios where the molecular landscape is large and diverse.

## Materials and methods

To overcome the challenge of suboptimal feature learning by deep neural networks on limited data, we explored an unsupervised learning approach. We aimed to develop a feature extractor capable of mapping the initial molecular feature space to a nuanced and informative subspace, thus improving the overall DDI side effect prediction performance.

Here are the three main biological intuitions (hypotheses) underlying the choice of a contrastive learning based approach. The first hypothesis (Hypothesis 1) states that by pre-training a molecular feature extractor using a contrastive learning approach on enumerated SMILES, the learned feature space will cluster drugs with similar molecular structures, indicating potential similarities in side-effect profiles. Since the scaffold is a structural framework representing the core molecular structure of a compound, while peripheral functional groups and substituents are ignored, molecules are more likely to be grouped based on their scaffold. Scaffolds have been shown to encode key aspects of biological activity [28]. This is because the core structure of a molecule often plays a crucial role in determining its activity. At the same time, peripheral functional groups and substituents can modulate the activity or influence pharmacokinetic properties. By focusing on the scaffold, researchers can compare the biological activity of different compounds with the same core structure, even if they have different functional groups or substituents. Scaffold-based drug design is an important approach in drug discovery, especially in cases where the exact mechanism of action of the new molecule is unknown or complex. Researchers develop new compounds with similar or improved properties by identifying and modifying key structural features of known scaffolds [28].

The second hypothesis is that pre-training using SMILES enumeration to generate multiple SMILES strings for each molecule will increase the diversity of the data (Hypothesis 2a) and further improve the robustness and performance of our drug–drug interaction side effect prediction model (Hypothesis 2b). SMILES enumeration generates different canonical SMILES strings for the same molecule by systematically enumerating all possible arrangements of atoms and bonds in the molecule. This data augmentation technique, commonly used in cheminformatics, has improved the robustness and performance of machine learning models [29].

The third hypothesis (Hypothesis 3) states that the pre-trained stable 'core' molecular representation acquired during the contrastive learning phase for predicting side effects of drug–drug interaction would improve the generalization of the model to new chemical compounds compared to traditional and non-pre-trained molecular features. By pre-training on a large unlabeled molecular dataset, the learning goes beyond the supervised drug–drug interaction dataset. It covers compounds outside the dataset without the need for additional labeled drug pairs. The larger coverage of chemical space compared to the supervised setting also improves the model’s ability to generalize to new molecules.

In this paper, we present a two-step framework for predicting the side effects of molecules. In the first step, we train a 1-D convolutional neural network (CNN) using a contrastive learning approach that allows us to extract informative molecular representations. We use the pre-trained model as a feature extractor and then add new, fully connected layers for classifying the side effects. We trained only the newly added layers with the drug–drug interaction dataset. The main components of the approach are explained in more detail later.

## Datasets

### ChEMBL22

**Fig. 1:**
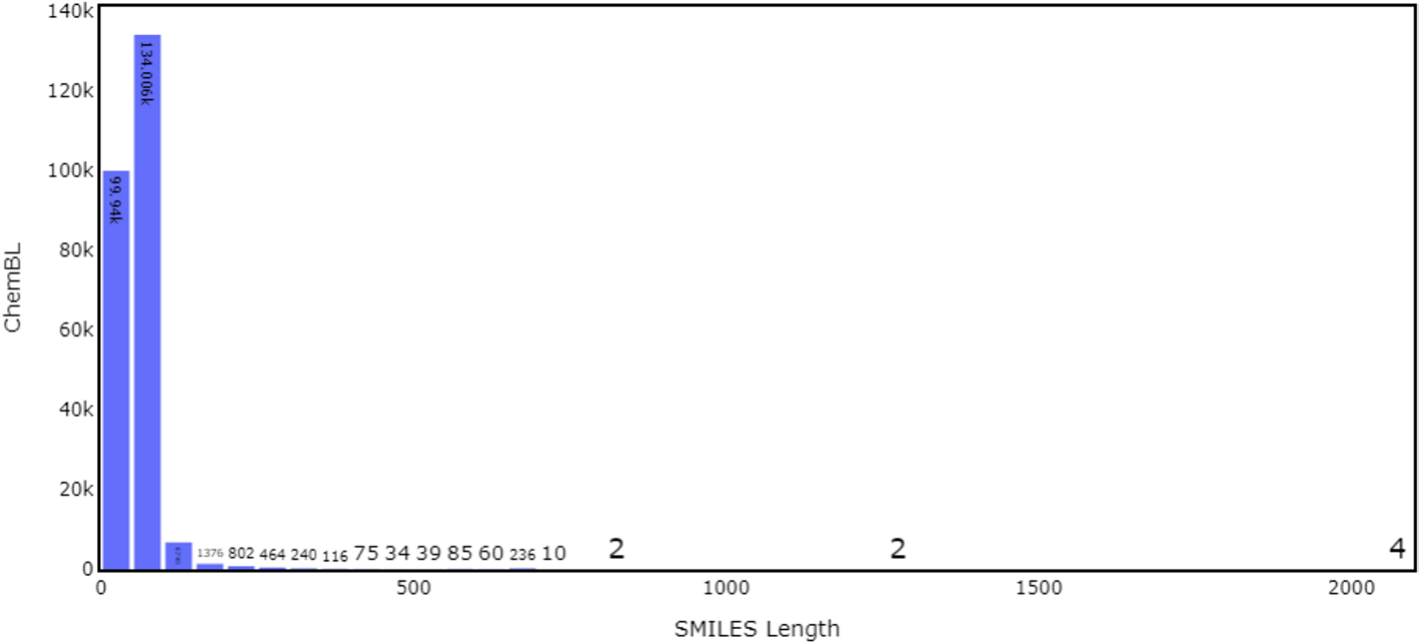
SMILES length distribution. SMILES length in ChemBL22 ranges from 7 to 2100 characters, with the largest SMILES sequence comprising 2100 characters and the smallest being seven characters long

We have obtained a dataset of drug-like molecules in SMILES format from the ChEMBL database (version 22.0). ChEMBL is a comprehensive bioactivity database with a large collection of unique chemical entities and a wide range of bioactivity measurements, such as binding, inhibition, and physiological effects. The database also contains information on drug targets and their interactions with small molecules, drug metabolism, and pharmacokinetic data. ChEMBL is widely used in drug discovery and development, bioinformatics, and computational biology research. Our dataset (version 22.0) consisted of 244,245 unique molecules (Fig. 1) and was downloaded from the DeepChem [30] MoleculeNet suite of datasets.

The SMILES were dynamically enumerated at each epoch using a Python script based on the cheminformatics library RDKit [31]. The atomic order of the molecule is randomized by converting it to molfile format and changing the atomic order. The molecule is then converted back to RDKit mol format, and a SMILES is generated using RDKit. The canonical SMILES option is set to false, so different orders of atoms can result in different SMILES. Some molecules can sometimes have numerous SMILES strings, which exceeds our requirements. To address this problem, we limited the number of SMILES strings to 50 based on our test experiments. Next, we tokenized the SMILES strings into a sequence of characters. We padded them to a fixed length using the SmilesToSeq vectorizer provided by DeepChem to ensure compatibility with the model architecture.

### Drugbank

**Fig. 2:**
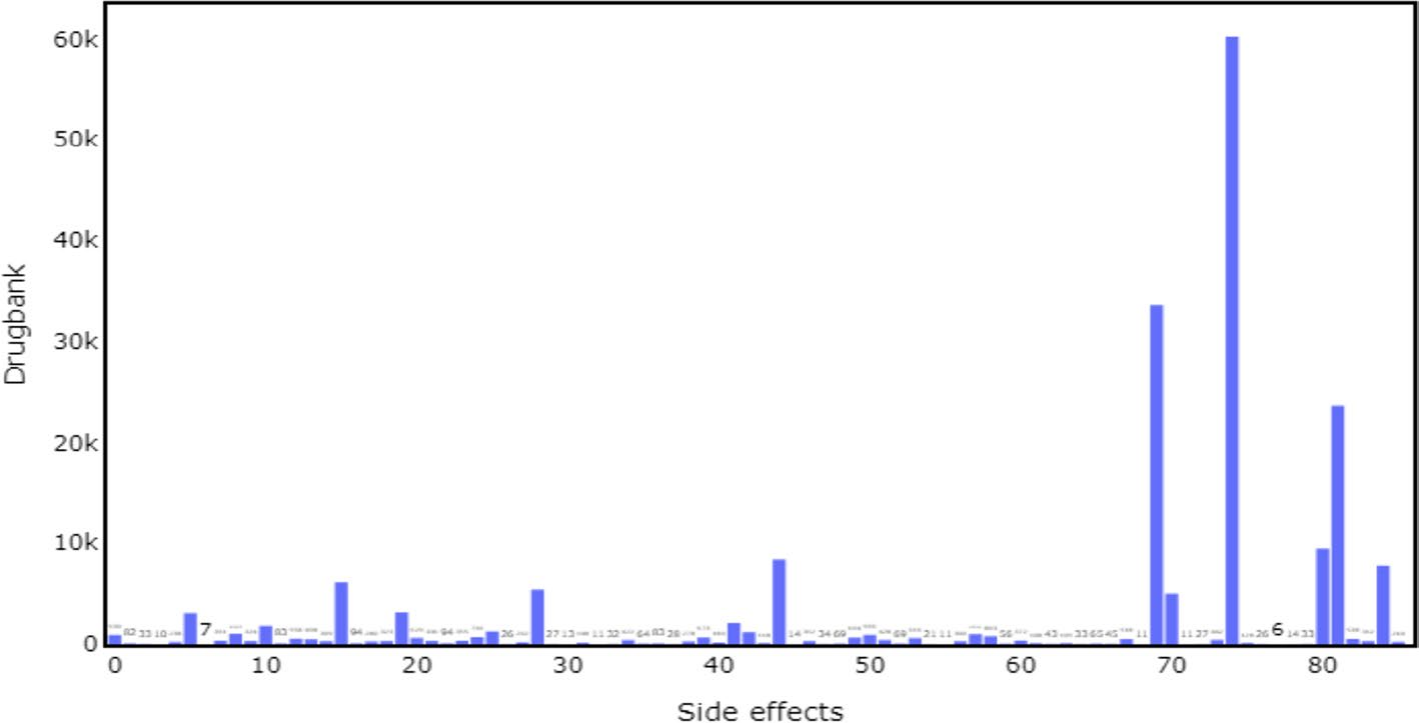
Distribution of Drugbank DDI side effects

Drugbank is a comprehensive online database containing drug targets, interactions, and metabolism information. It is sourced from FDA/Health Canada drug labels and primary literature and downloaded from Therapeutics Data Commons (TDC).^1^ It is a highly imbalanced dataset that provides detailed information on more than 191,808 drug–drug interactions involving 1706 drugs and 86 side effects. Figure 2 shows the distribution of side effects in Drugbank.

## Model architecture

### Contrastive learning of drug representation

#### Problem formulation

Consider a dataset of molecular structures represented by SMILES strings, denoted as $D = \left\{ {x_{1} , \, x_{2} ,..,x_{N} } \right\}$, where *N* is the number of molecules. The goal is to learn a feature extractor function *f* that maps each molecular structure $x_{i}$ to a feature space *z*_*i*_ = *f*(*x*_*i*_). Let *S*(*x*_*i*_) be the set of SMILES strings similar to *x*_*i*_, generated using SMILES enumeration. The contrastive learning objective is to maximize the similarity between the feature representations of positive pairs (*x*_*i*_, *x*_*j*_ ${ \in }S(x_{i} )$) and simultaneously minimize the similarity between the feature representations of negative pairs ($x_{i} ,x_{k} \notin S(x_{i} )$). Given a minibatch $B \in D,\left\| B \right\| = m$, we used the Noise-Contrastive Estimation with Information Maximizing Objective (InfoNCE) loss function [32]:

1 $${\mathcal{L}}_{{{\text{InfoNCE}}}}^{(i,j)} = - \log \frac{{\exp ({\text{sim}}(f(x_{i} ),f(x_{j} ))/\tau )}}{{\sum\nolimits_{k = 1}^{2m} {{ \nVdash }_{[k \ne i]} } \exp ({\text{sim}}(f(x_{i} ),f(x_{k} ))/\tau )}}$$

where ${ \nVdash }_{[k \ne i]}$ is an indicator function: 1 if $k \ne i$, 0 otherwise, ${\text{sim}}(.,.)$ is a cosine similarity function, and $\tau$, a temperature parameter that controls the sharpness of the similarity function. The cosine similarity function for vectors $a$ and $b$ is calculated as follows:

2 $${\text{sim}}({\mathbf{a}},{\mathbf{b}}) = \frac{{{\mathbf{a}} \cdot {\mathbf{b}}}}{{\left\| {\mathbf{a}} \right\| \cdot \left\| {\mathbf{b}} \right\|}}$$

where $\left\| {\mathbf{.}} \right\|$ represents the Euclidean norm. The maximum cosine similarity possible is 1, while the minimum is − 1. This formulation encourages the model to learn a representation such that similar molecules are brought close together in the feature space while pushing dissimilar molecules apart. This is beneficial for tasks such as molecular properties prediction or drug discovery.

We have adapted the SimCLR2 architecture, which was originally developed for images, so that it can effectively encode SMILES strings. The base encoder network ${\text{e}}(.)$ is an embedding layer followed by a series of conv1D layers and is responsible for extracting a representation vector $h$ from the augmented SMILES. The embedding layer maps each atom in the SMILES to a continuous vector of size 116, using a vocabulary of 148 elements that includes all atoms of the periodic table and some special characters, such as @ and \, which are used to construct the SMILES. We use the Rectified linear unit activation function (ReLU), and dropout and batch normalization layers are placed between the convolutional layers of the neural network layers.

**Fig. 3:**
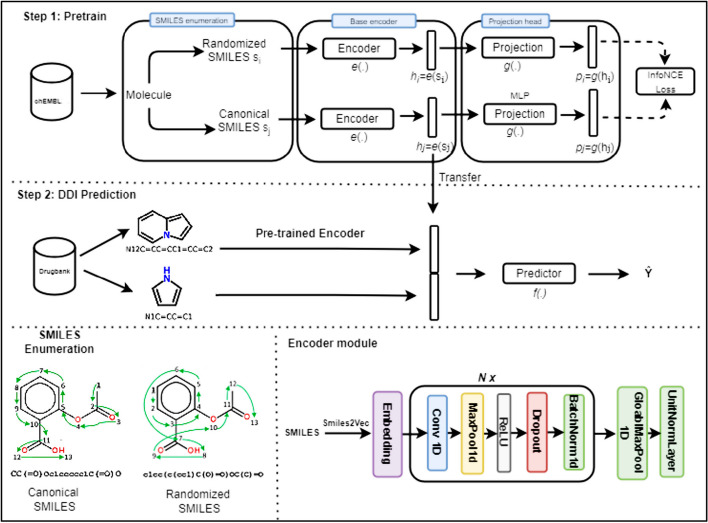
Overview of SMR-DDI. Step 1: The molecules are sampled from ChEMBL22, and SMILES enumeration is applied to generate a randomized view of the molecule. A base encoder network ${\text{e}}(.)$ and a projection head ${\text{g}}(.)$ are trained to maximize the similarity between the canonical and randomized SMILES using the InfoNCE contrastive loss. Step 2: After the training process is completed, the representation $h$ is transferred for DDI prediction. The latent features of each drug pair are combined to create a vector that is fed into a feed-forward neural network to predict DDIs

The projection head ${\text{g}}(.)$ is a fully connected neural network with two layers that map the representation $h$ to a space where we apply the contrastive loss. The architecture is shown schematically in Fig. 3. For each training iteration, we created a second view for each canonical SMILES in our batch using SMILES enumeration. The two SMILES are then tokenized into sequences, padded with *smilestoseq*, and fed into the encoder to obtain a 1D feature vector to which the projection head is applied. The output features of the two augmented SMILES are then trained to be close to each other and different from the feature vector of the remaining SMILES in the batch.

#### Model training and evaluation

We split the ChEMBL dataset into training and validation sets in a ratio of 80:20. Since we want the molecules in the validation set to be as diverse as those in the training set, we used DeepChem's MaxMin splitting strategy. The MaxMin algorithm is a common technique for selecting different subsets of molecules from a larger pool. It selects a group of dissimilar molecules representing the full range of chemical space in the larger pool.

To train the model, we used the AdamW optimizer with a cosine annealing learning rate scheduler. The initial learning rate was set to 1e−3, and the weight decay was set to 1e−6. We set the maximum number of epochs to 50. The model was trained on a single NVIDIA GeForce GPU with a batch of size 512. We used PyTorch Lightning to train the model with DataParallel in a distributed manner. We monitored the training progress with TensorBoard and saved the best model based on the validation loss. We randomly searched multiple architectures and selected the architecture with the lowest validation loss.

### Prediction of drug–drug interaction

#### Problem formulation

Our goal is to build a machine learning model to predict the side effects of drug–drug interactions. We formulated the problem as a multiclass classification task where each drug pair is associated with one side effect. Given a set of *n* drug pairs $X: = \{ (x_{1}^{1} ,x_{1}^{2} ), \ldots ,(x_{n}^{1} ,x_{n}^{2} )\left| {x_{i}^{1} \in {\mathcal{X}} \, \,{\text{and }}\,x_{i}^{2} \in {\mathcal{X}}\} } \right.$, a set of side effect labels, $Y: = \{ y_{1} , \ldots ,y_{n} \left| {y \in {\mathbb{N}}} \right.\}$ and the molecular feature space $\chi$, the goal is to learn a function $f$ parametrized by $\theta$ that maps a drug pair $x_{i} : = (x_{i}^{1} ,x_{i}^{2} )$ to a discrete probability distribution over all possible side effects $y \in Y$. Specifically, we minimized the negative log-likelihood loss between $f(x_{i} ;\Theta )$ and $y_{i}$ for each pair $x_{i}$. The Negative log-likelihood over $X$ is defined as:

3 $${\mathcal{L}}\left( {\theta \, \left| X \right.} \right) = - \frac{1}{n}\sum\limits_{i = 1}^{n} {\log } P(Y = y_{i} \left| {x_{i} ;\theta } \right.) = - \frac{1}{n}\sum\limits_{i = 1}^{n} {y_{i} } \log \,f(x_{i} ;\theta )$$

where $P(Y = y_{i} \left| {x_{i} ;\theta } \right.)$ is the predicted probability distribution over $Y$ given $x_{i}$ and model parameters $\theta$, and $y_{i}$ is the true label.

#### Model training

After training with contrastive learning, we removed the projection head ${\text{g}}(.)$ and used ${\text{e}}(.)$ it as a pre-trained feature extractor. We used a pre-trained 1D-CNN encoder to encode the SMILES strings of drugs from the Drugbank. We then trained a fully connected neural network to predict the side effects associated with each drug pair. For each drug pair in Drugbank, the pre-trained molecular representations were concatenated and fed to the classifier. The number of layers and the size of each layer are optimized using Optuna, and only the best model is saved.

We trained the classifier with a batch size of 256 over 200 epochs and selected only the best epoch to perform inference. We used the same optimizer and learning rate scheduler as before. We split the Drugbank dataset into training, validation, and test sets in an 80:10:10 ratio to evaluate the performance of the model. We repeated this process for five random seeds for a more robust estimate. We used a stratified sampling strategy to ensure that the distribution of classes remained consistent across partitions.

#### Evaluation schemes

This study evaluated DDI prediction tasks based on three experimental settings:

- Random split: prediction of unobserved interaction types between known drugs (Task 1). After deployment, the model will only be exposed to drugs seen during training, even if the pairs the model is asked about are unseen. This is the classical train-test split scenario.
- One-unseen split: prediction of interaction types between known drugs and new drugs. This scenario is relevant when using our DDIs models to predict safety liabilities associated with taking recently approved drugs with existing ones. (Task 2)
- Both-unseen split: prediction of interaction types between new drugs (task 3). The new drugs in the corresponding task are missing in the training set but are present in the test set. This scenario helps quantify how well the models can utilize existing DDIs models to explore new drug combinations.

**Fig. 4:**
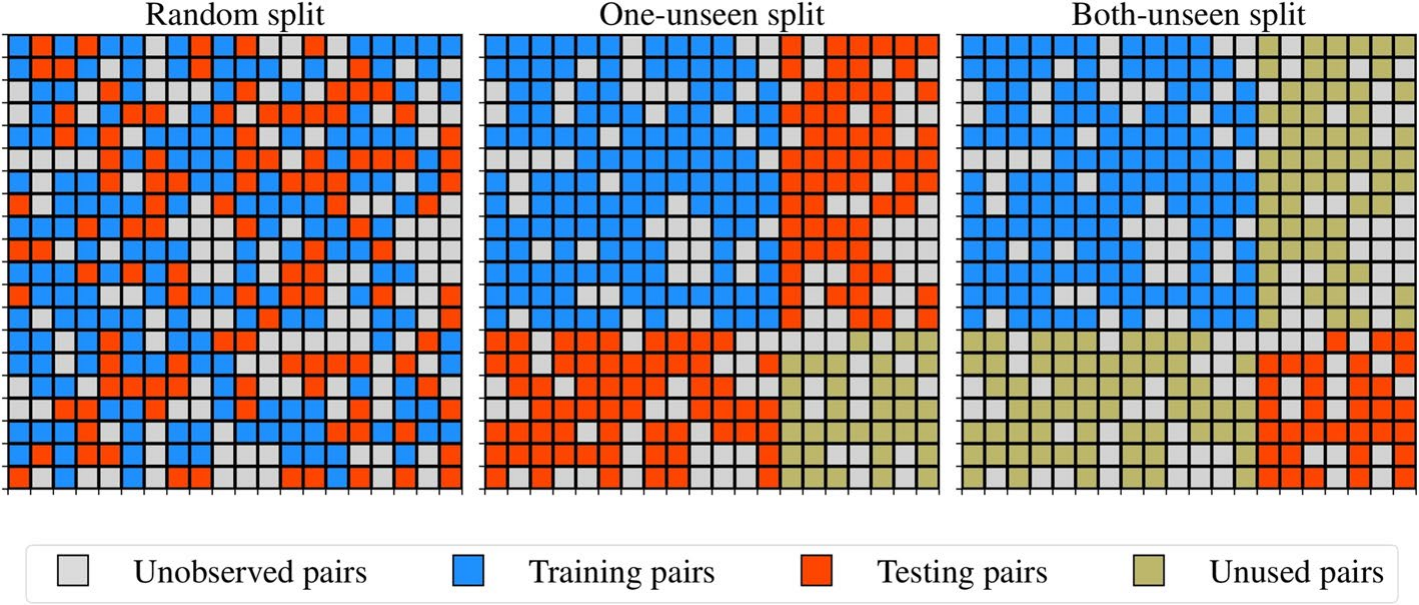
Evaluation schemes for DDIs models. Left: random splitting strategy; center: one-unseen splitting strategy; right: both-unseen splitting strategy. Combining the strategy on the right and center is recommended to avoid unnecessary data waste. One unseen and both-unseen share the same training examples

The three experimental settings help to get a complete overview of how well a model can predict the side effects of drug–drug interactions. Figure 4 summarizes all three evaluation schemes.

#### Metrics

We used the area under the receiver operating characteristic curve (AUC-ROC), the area under the precision-recall curve (AUPRC), accuracy (ACC), F1-score, Precision, and Recall as our performance metrics. The AUC-ROC measures the trade-off between the true positive rate (TPR) and the false positive rate (FPR) and is computed as the area under the ROC curve. The AUPRC computes the area under the precision-recall curve and provides a measure of the trade-off between precision and recall. Accuracy (ACC) is a standard performance metric that measures the percentage of correctly classified instances. The F1-score is the harmonic mean of precision and recall, which considers false positives and negatives. Precision is the ratio of true positives to the sum of true positives and false positives. Recall is the ratio of true positives to the sum of true positives and false negatives. Together, these performance metrics provide a comprehensive assessment of the performance of our model. As the dataset is highly imbalanced, we use the weighted version of these metrics to account for the distribution of each side effect. Only AUC-ROC and AUPRC are macro metrics.

## Results and discussion

The experiments in the Results section can be divided into two groups. The first three experiments investigate how useful a feature space based on scaffolds can be for predicting the side effects of drug–drug interactions. The remaining experiments evaluate the quality of the feature space and show the results of predicting the side effects of drug–drug interactions.

### Frequent pattern mining: exploring scaffold combinations and association rules

The first experiment uses frequent pattern mining techniques to explore scaffold combinations and association rules within the Drugbank dataset, which contains extensive information on drug interactions and side effects. We started by extracting drug pairs and their associated side effects from Drugbank and used DeepChem^2^ to determine the scaffolds of each molecule. We identified the most frequent combinations by applying the FP-Growth algorithm with a minimum support threshold of 1%. This dataset of 191,808 molecule combinations yielded 964 scaffold families and 93,681 unique scaffold pair combinations (Additional file 1: supplementary file 1a). Interestingly, 75% of the scaffold combinations occurred only once. As expected, the benzene ring (canonical SMILES c1ccccc1) was the most frequently observed scaffold, with the most recurrent combination of scaffolds being (c1ccccc1, c1ccccc1). We derived 59 combinations from the FP growth analysis, with the most frequent combination having a support value of 28% (Additional file 2: supplementary file 1b). Using this set of 59 combinations, we generated a comprehensive collection of association rules (Additional file 3: supplementary file 1c). These rules provided information about the likelihood of specific side effects occurring with certain combinations of drugs or interactions between scaffolds. We used support, confidence, and lift metrics to score the rules. For example, the association rule with the highest confidence level (62%) suggests that if ‘*#Drug1 may decrease the antihypertensive activities of #Drug2*,’ one of the molecules contains at least one benzene ring. This is consistent with another rule in the database, which states that if ‘c1ccccc1’ is present, then ‘#Drug1 may increase the hypotensive activities of #Drug2.’ Although the benzene ring is commonly used in pharmaceuticals due to its desirable physicochemical properties, such as lipophilicity, planarity, and ability to interact with certain receptors or enzymes in the body, there is no direct evidence in the literature linking benzene to hypotensive activities. Therefore, this rule probably stems from the fact that the most frequent combination of scaffolds is the pair of benzene. Thus, it is important to note that the imbalance in the distribution of scaffold families within the dataset may introduce bias into the generated rules. Furthermore, limitations of the dataset, such as potential data incompleteness, need to be considered when interpreting the results.

### Scaffold interaction profiles analysis

In a second experiment, we attempted to mitigate potential frequency biases and investigate similarities between the scaffolds by examining their interaction profiles. We assessed the presence or absence of interactions within the dataset for each pair of scaffolds. We created a binary interaction profile matrix in which each cell (i, j) indicates whether scaffold i interacts with scaffold j (0 for no interaction, 1 otherwise). Each row is a binary vector describing the interaction partners for each scaffold. By using a binary vector instead of counting the number of interactions between each scaffold, the bias in the rules due to imbalance in the dataset was removed. This matrix also answers whether we can infer potential interaction partners for a molecule based on its scaffold. It also allowed us to compare each scaffold based on its interaction partners and cluster scaffolds with similar interaction profiles.

**Fig. 5:**
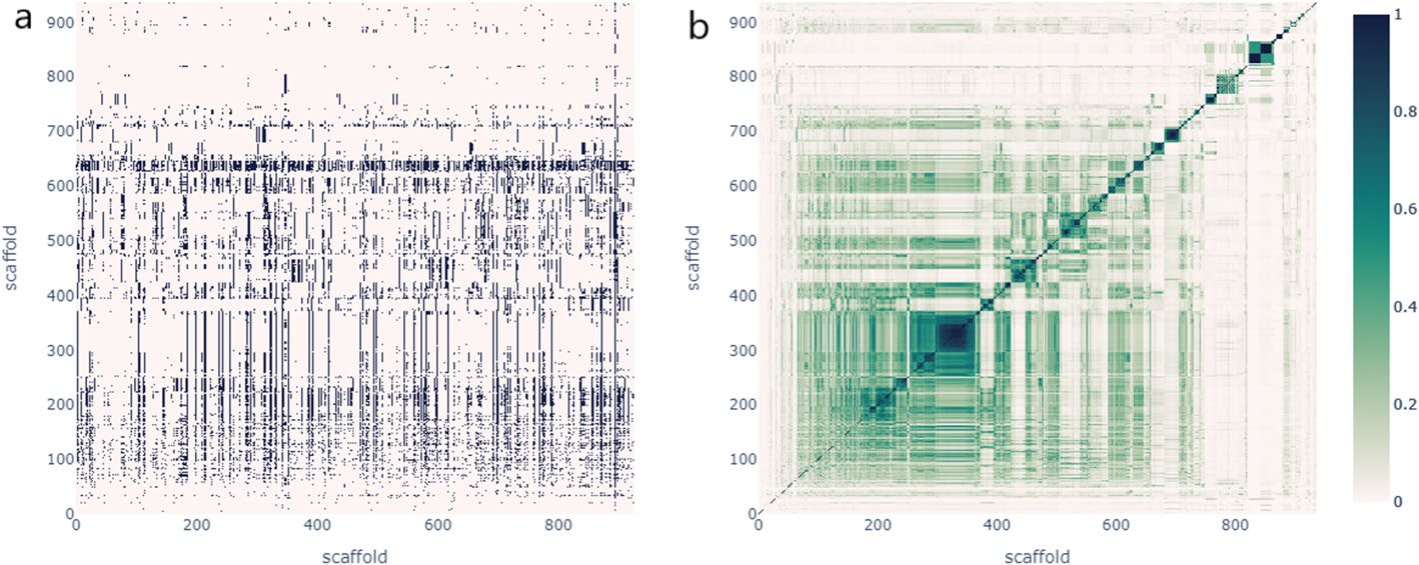
Scaffold Interaction Profiles and Cluster Analysis. **a** Binary interaction profiles matrix: Each cell represents the presence (black pixel) or absence (white pixel) of interaction between scaffolds. **b** Scaffold-Scaffold similarity matrix: Scaffolds are clustered based on the similarity of their interaction profiles. Darker cells indicate higher similarity between scaffold interaction profiles

The analysis resulted in a matrix with 937 rows and 926 columns, representing the number of unique scaffolds in the columns for drug 1 and 2, respectively (Fig. 5a). We found that the families of molecules had a relatively low number of interaction partners, with 50% of the scaffolds interacting with 79 or fewer scaffolds of the 926 candidates. The most represented scaffold, c1ccccc1, had the highest number of interaction partners (686 out of 926, representing 74% of possible interaction partners). In contrast, some families had only one interaction partner (1 out of 926). Although some family combinations were rarer than others, it is not clear whether these families inherently have fewer interaction partners or whether this is simply due to the limitations of the dataset.

**Fig. 6:**
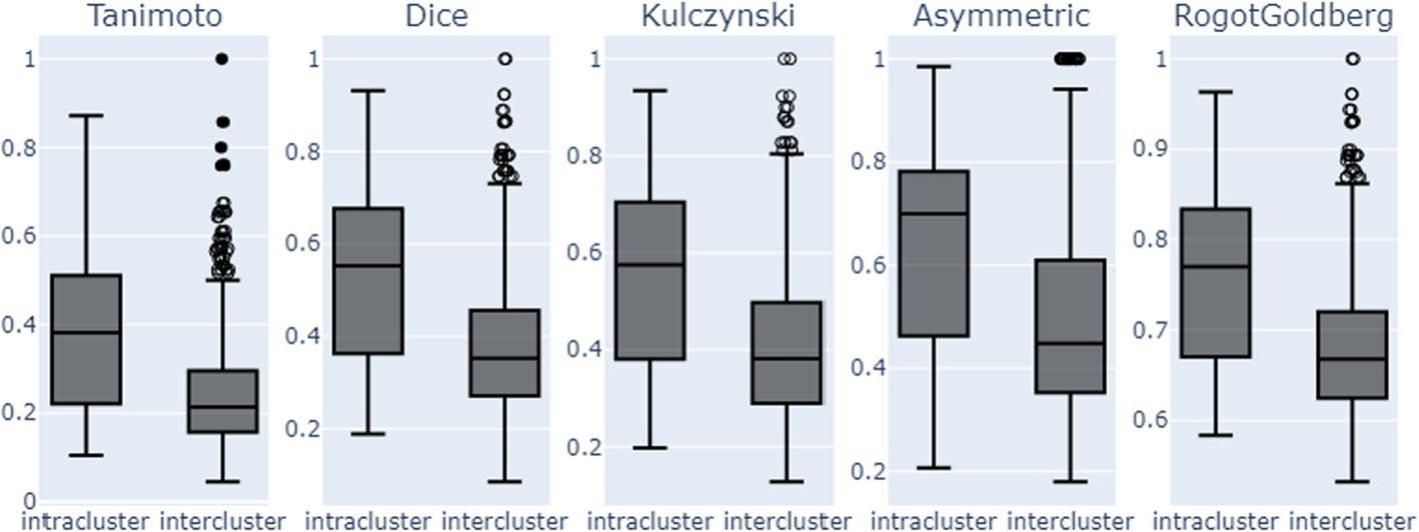
Structural Similarity Analysis of Scaffold Clusters formed based on scaffold interaction profiles. Intracluster similarity: Average pairwise similarity among scaffolds within the same cluster. Intercluster similarity: Average pairwise similarity between scaffolds of different clusters

We used the algorithm *fclusterdata* from the SciPy library with default parameters and the Jaccard similarity metric to cluster the scaffolds based on their interaction profiles (Fig. 5b). We identified 417 scaffold clusters, with the smallest cluster containing one scaffold and 75% of the clusters having one or two scaffolds (Additional file 4: supplementary file 2). The largest cluster consisted of 30 scaffolds. To complete our analysis, the resulting clusters were examined to determine whether these groups exhibit similarities from a structural perspective. In particular, we focused on clusters whose members shared more than 90% of their interaction profiles. Even though Tanimoto is the best known similarity metric, as recommended by [33], we decided to explore other metrics that could provide valuable insights for our analysis. We used the RDKit library to investigate four other Tanimoto variants: RogotGoldberg, Asymmetric, Kulczynski, and Dice. Like Tanimoto, these variants measure the proportion of shared bits between two fingerprints out of the total number of bits set in both fingerprints, with the main difference being how they assign weights to the shared bits (Fig. 6).

The different similarity metrics indicated that scaffolds with similar interaction profiles shared specific structural characteristics but were not identical. Families within the same cluster were more similar to each other than to others. However, the interpretation of similarity scores depends on the specific application and context. For instance, in drug discovery, unlike virtual screening applications that require higher similarity scores to identify potential leads confidently, a Tanimoto similarity score ≥ 0.3, in some cases, is considered sufficient to indicate structural similarity between molecules [34–37].

### Scaffold side effects profiles analysis

The same (previous) experiment was repeated by generating a binary vector of side effects for each scaffold to assess the relationship between scaffolds and side effects. We wanted to analyze the profiles of side effects associated with each scaffold and investigate whether it is possible to derive a potential list of side effects from a scaffold or, conversely, whether we can derive a list of candidate scaffolds from a side effect. Similar to the previous experiment, we compared scaffold groups with similar side effect profiles to determine their structural similarities. We also compared side effects with similar scaffold profiles (list) to determine if they are biologically related.

**Fig. 7:**
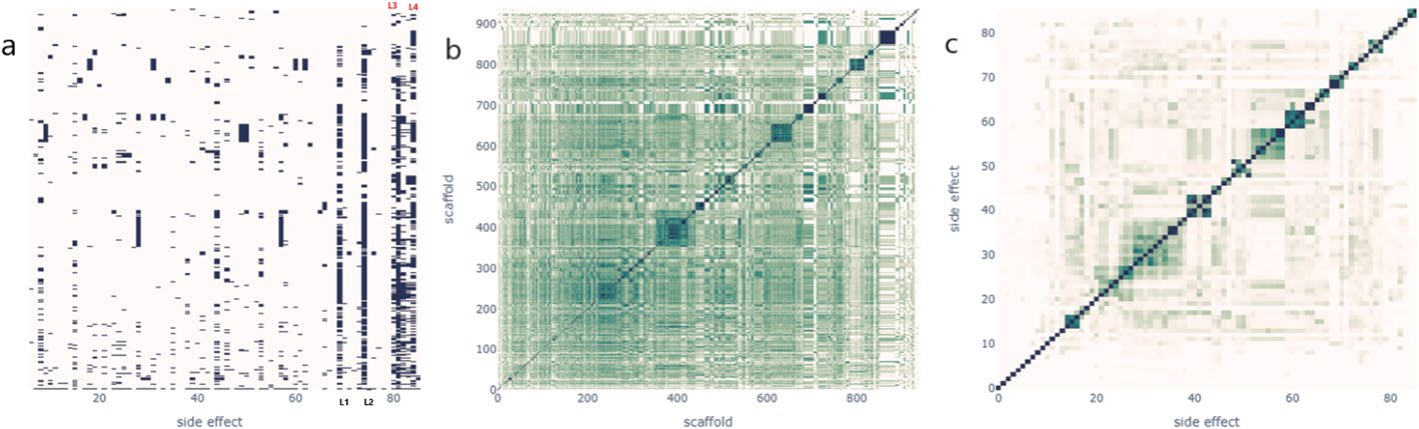
Scaffold side effects profiles and Cluster Analysis. **a** Binary interaction profiles matrix: Each cell represents the presence (black pixel) or absence (white pixel) of association side-effect: scaffolds. The four plain black lines are named L1 up to L4, respectively. **b** Scaffold-Scaffold similarity matrix: Scaffolds are clustered based on the similarity of their side effect profiles. Darker cells indicate higher similarity between scaffold interaction profiles. **c** Side effect—Side effects similarity matrix: Side effects are clustered based on how similar the list of scaffolds that cause them are. Darker cells indicate higher similarity between side effects scaffold profiles

Specifically, we generated a binary side effect profile matrix for scaffolds, where each row is a scaffold, and each column is a side effect (filled with 1 when the side effect is reported for the scaffold, 0 otherwise). The resulting matrix has 939 rows and 86 columns (Fig. 7a). Approximately 75% of the scaffolds exhibited 1 to 9 side effects, representing around 9% of the total side effects, with 7% having only one out of 86 side effects. The scaffold “c1ccccc1” was associated with 61 out of 86 side effects, indicating its wide range of side effects. We observed that some side effects, such as L1–L4 (Additional file 5: supplementary file 3a), were more challenging to predict in a leave-one-drug-out setting as they appeared in most families of molecules.

**Fig. 8:**
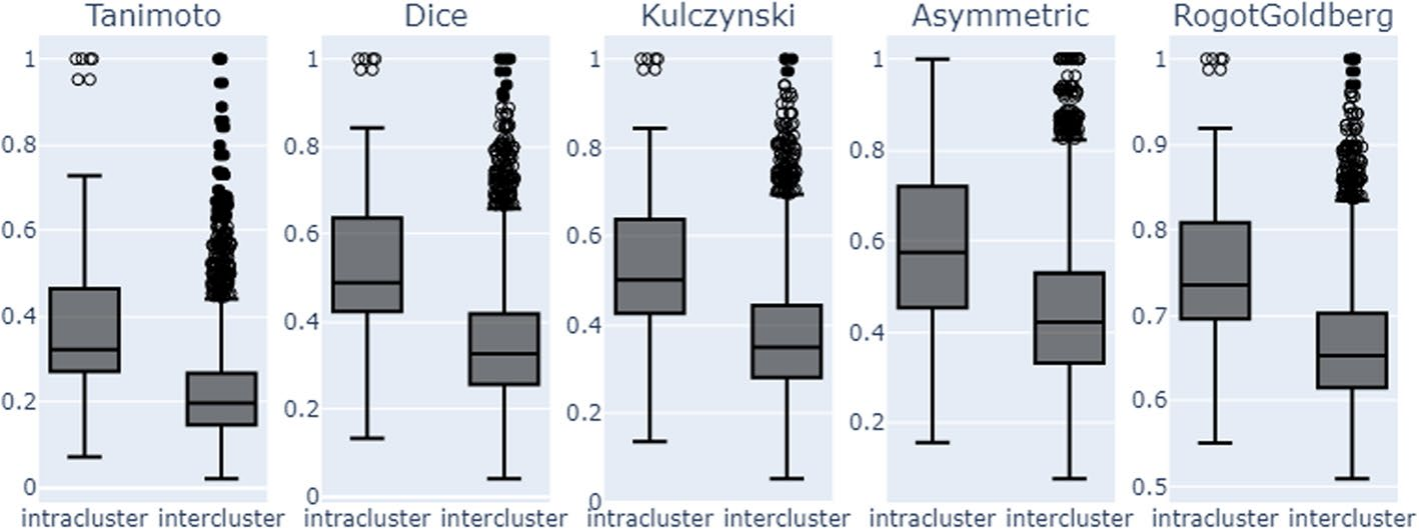
Structural Similarity Analysis of Scaffold Clusters formed based on their side effects profiles

We also calculated the pairwise similarity between scaffolds based on their side-effect profiles and used the same clustering algorithm to cluster scaffolds with similar side-effect profiles. This matrix revealed the presence of 364 clusters among the scaffolds (Fig. 7b). The clusters varied in size, with the smallest containing only one scaffold and 75% having one or two scaffolds. The largest cluster consisted of 45 scaffolds. Molecular similarity analysis of the clusters (Fig. 8) showed that families of molecules with similar side effect profiles, although not identical, shared certain structural features. However, knowing the scaffold of a molecule in our dataset significantly reduced the range of possible side effects. On average, we went from an initial space of 86 side effects to approximately nine side effects per scaffold. This could be very useful for predicting the potential side effects of new or unseen compounds that belong to one of these clusters.

We further investigated side effects with similar scaffold profiles and obtained a second distance matrix (similarity) of size 86 × 86, which yielded 38 clusters (Fig. 7c). The smallest cluster contained two side effects, 75% contained up to three side effects, while the most significant cluster contained seven side effects. We also identified six pairs of side effects that shared the same scaffold profiles (Additional file 6: supplementary file 3b). When inspecting the side effect clusters, we discovered that the same list of scaffolds frequently caused biologically related side effects. For example, sedative drugs could also exhibit serotoninergic activities [38, 39]. Benzodiazepines are sedative drugs shown to modulate serotoninergic activity in the brain. Similarly, selective serotonin reuptake inhibitors (SSRIs), primarily used for their serotoninergic activity, can also have sedative effects due to their primary mechanism of action.

Overall, the previous experiments have highlighted the need for an expressive feature space capable of classifying molecules based on their scaffolds and capturing the subtleties of individual molecules. The following steps involve assessing if the proposed deep learning architecture can learn such a space.

### Visualization of molecular representations

**Fig. 9:**
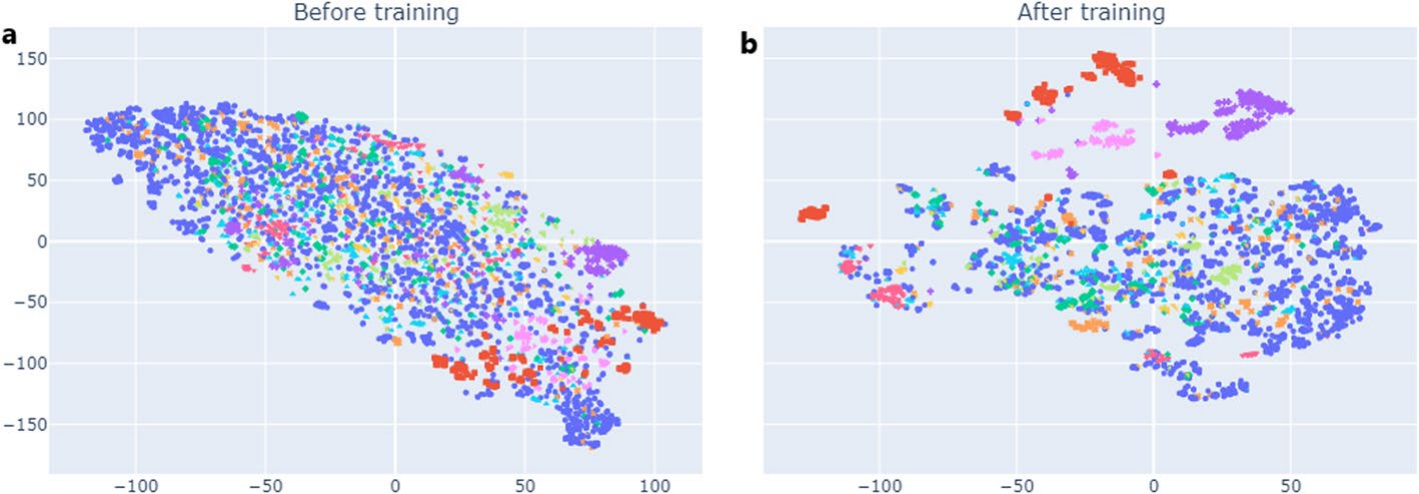
t-SNE Visualization of Molecular Representations Learned by SMR-DDI. The visualization includes 5000 randomly selected molecules from the ChEMBL22 validation dataset. Each molecule is color-coded based on its scaffold

We aimed to validate the learned latent space obtained by pretraining the SMR-DDI model on the ChEMBL22 dataset, which comprises a vast collection of unique chemical entities and a wide range of bioactivity measurements, such as binding, inhibition, and physiological effects. Using t-distributed stochastic neighbor embedding (t-SNE), we randomly selected 5000 molecules from ChEMBL22 and projected the 262-dim latent space learned by SMR-DDI into a 2D representation. t-SNE is a non-linear dimensionality reduction technique that preserves pairwise similarities between data points while mapping them from a high to a lower-dimensional space. It is particularly effective in revealing non-linear structures in data that are challenging to discover using linear methods such as PCA. The hyperparameters used for t-SNE were n_components = 2, perplexity = 30, and n_iter = 5000. Each molecule in the 2D space was color-coded based on its molecular family (Fig. 9). The results showed that the learned latent space enabled the separation of molecules based on their scaffolds. However, we found that certain families of molecules are too closely grouped.

**Fig. 10:**
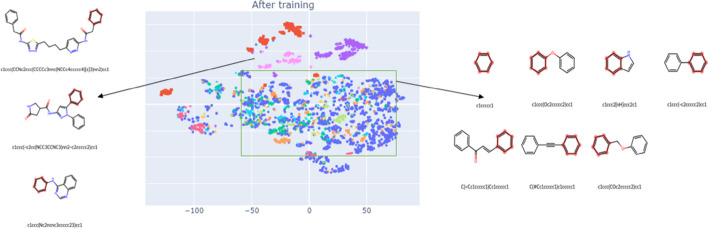
Molecular Validation of Scaffold Relationships Learned by SMR-DDI

Figure 10 examines then whether the distance between the different clusters of scaffolds learned from SMR-DDI is representative of their structural similarity. In particular, the analysis compares the molecular structures of drugs with closely clustered scaffolds (green box) with those with distinctly clustered scaffolds (outside the green box). When the molecular structures were analyzed with RDKit, we found that the molecules outside the green box had distinct structural patterns. In contrast, the molecules inside the green box were very similar, with the core structure consisting of two benzene rings with different bonds. For example, molecules such as 1ccc(COc2ccccc2)cc1 and c1ccc(–c2ccccc2)cc1 are very similar, except for the presence of a methoxy group in one molecule and a direct carbon–carbon (–) bond between the benzene rings in the other. This observation confirmed the correct positioning of the molecules based on their structural similarities. Furthermore, an LDA analysis of the green box molecules confirmed that there is a projection that maximizes the distance between different scaffolds even if they are structurally very similar (Additional file 7: Supplementary Fig. S1).

**Fig. 11:**
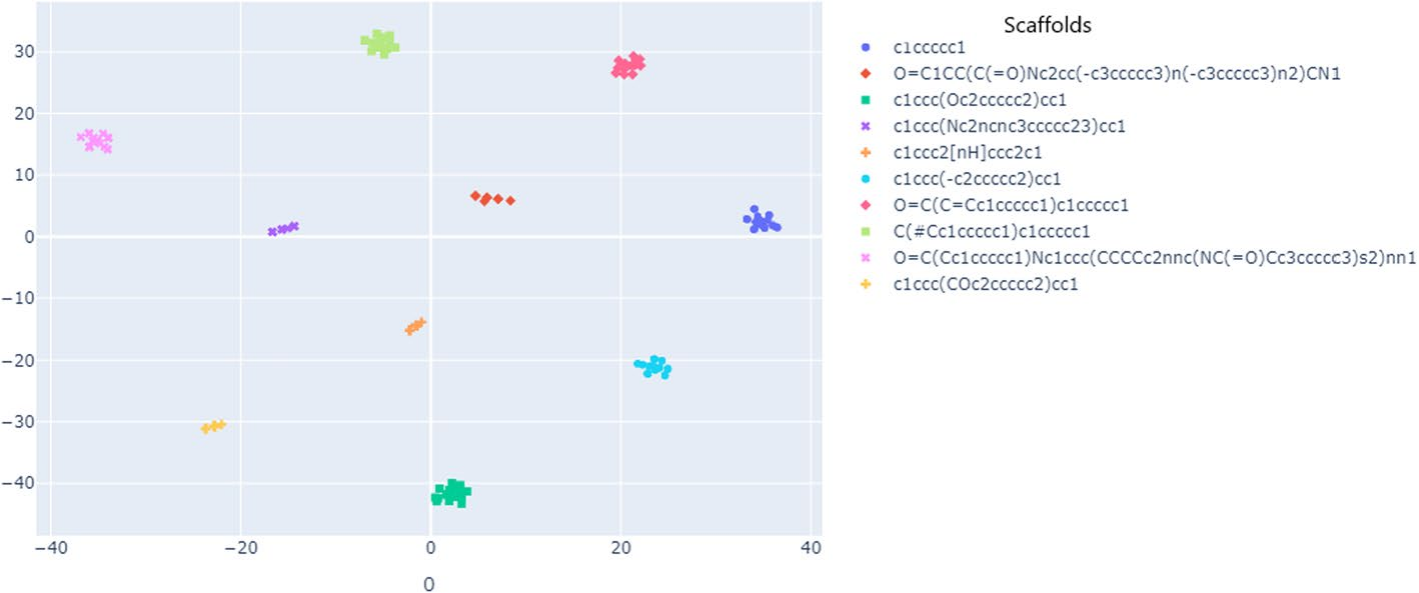
t-SNE visualization of the canonical and randomized SMILES feature space. One hundred canonical SMILES and their corresponding ten randomized SMILES per canonical SMILES

Finally, we randomly selected 100 SMILES (molecules), generated ten randomized views for each molecule, and visualized the feature space with t-SNE (Fig. 11). We found that the canonical and randomized SMILES were close in the generated vector space. This closeness suggests that SMR-DDI has adequate expressive power to capture the relationships between canonical and randomized views. The learned feature space can effectively represent the structural similarities and differences of the molecules. The network had learned close representations for molecules with similar molecular structures even without labeling information during training, indicating the model's ability to capture intrinsic connections between molecules. These results confirmed our first hypothesis (Hypothesis 1), which stated that pre-training a molecular feature extractor using a contrastive learning approach on enumerated SMILES would result in a feature space that clusters drugs with similar molecular scaffolds.

### DDI prediction: handling imbalanced data

To quantify the quality of the pre-trained space, we trained a deep neural network to predict Drugbank side effects using the pre-trained model (contrastive learning framework) as a feature extractor. We added additional layers for side effect classification and trained only the new ones on the new dataset. We used negative log-likelihood loss as a loss function and a batch size of 256. We optimized hyperparameters such as the number of layers, dropout, and learning rate optimization using Hyperopt. We evaluated not only the performance in conventional prediction schemes, such as the types of interactions not observed between known drugs (Task 1), but also the robustness of generalization in two other experimental settings. The first setting involved evaluating the prediction of known drug interactions (Task 1), the second one the prediction of interactions between known and new drugs (Task 2), and the third one the prediction of interactions between new drugs (Task 3). The new drugs in the corresponding tasks were absent from the training set but present in the test set. The dataset used was Drugbank and is heavily imbalanced. To address the class imbalance, we tested different approaches during training, such as balancing batches, using class weights, and a weighted random sampler from PyTorch (WRSP). Balancing the batches ensured an equal number of examples per class in each batch, with the batch size set to 256. Class weights involved sampling each class randomly with replacement, where the probability of sampling a class was inversely proportional to its frequency in the dataset. This strategy favored the minority classes, as they had a higher chance of being sampled. The weighted random sampler in PyTorch allowed random data sampling, with the sampling probability proportional to the weight of each sample. To provide a comprehensive evaluation of the prediction model's performance, we reported in Table 1 metrics that accounted for class imbalance, such as precision, recall, weighted F1 score, area under the receiver operating characteristic curve (AUC-ROC), and area under the precision-recall curve (AUPRC). In addition, confusion matrices were presented (Additional file 8: Supplementary Fig. S2). We obtained the best results using balanced batches or the weighted random sampler from PyTorch. Although the results of these two samplers were very similar, the balanced batch strategy performed slightly better. As we hypothesized earlier, L1 to L4 were more challenging to predict in tasks 2 and 3 (Additional file 8: Supplementary Fig. S2). We thus used balanced batches for all of our experiments.

**Table 1:** Performance of different sampling strategies

	AUPRC	AUROC	ACC	F1	Precision	Recall
*Task 1*						
BALANCED_BATCH	**0.897**	**0.991**	**0.912**	**0.912**	**0.913**	**0.912**
CLASS_WEIGHT	0.710	0.971	0.305	0.214	0.398	0.305
PyTorch WEIGTHED RANDOM SAMPLER	**0.868**	**0.990**	**0.862**	**0.862**	**0.866**	**0.862**
*Task 2*						
BALANCED_BATCH	***0.420***	**0.900**	**0.537**	**0.508**	**0.525**	**0.537**
CLASS_WEIGHT	0.390	**0.886**	0.237	0.180	0.433	0.237
PyTorch WEIGHTED RANDOM SAMPLER	**0.417**	0.878	**0.530**	**0.500**	**0.519**	**0.530**
*Task 3*						
BALANCED_BATCH	0.170	**0.731**	**0.299**	**0.264**	**0.283**	**0.299**
CLASS_WEIGHT	**0.205**	**0.756**	0.174	0.161	0.250	0.174
PyTorch WEIGHTED RANDOM SAMPLER	**0.175**	**0.730**	**0.299**	**0.255**	**0.269**	**0.299**

The best results are in bold

### Comparison with baselines

We conducted a performance comparison of our pre-trained feature space with several well-known feature spaces (molecular representations) from the literature, including:

- ECFP (radius 2, nbits = 2048): Extended-connectivity fingerprints (ECFPs) are a family of circular fingerprints designed for molecular characterization, similarity searching, and structure–activity modeling. They use a predefined set of structural groups called circular substructures. These substructures are circular fragments of a molecule of a certain radius and can be considered topological features that capture the connectivity of atoms within the molecule [40].
- Maccs: MACCS keys are 166-bit 2D structure fingerprints commonly used to measure molecular similarity. They described the presence of key features in molecular graphs [41].
- ChemGPT: is a transformers model for generative molecular modeling, pre-trained on the PubChem10M dataset. ChemGPT (1.2B params) has 1.2B params, while ChemGPT (4.7 M params) has 4.7 M params [42].
- ChemBERTa is a pre-trained language model for molecules based on (Ro)BERT(a) trained on PubChem 77 M compounds. The MTR version was pre-trained using a multitask regression objective, while the MLM version was pre-trained using a masked language modeling objective [43].
- gin_supervised_masking: GIN neural network model pre-trained with masked modeling on molecules from ChEMBL [44].
- gin_supervised_infomax: GIN neural network model pre-trained with mutual information maximization on molecules from ChEMBL [44].
- gin_supervised_edgepred: GIN neural network model pre-trained with supervised learning and edge prediction on molecules from ChEMBL [44].
- gin_supervised_contextpred: GIN neural network model pre-trained with supervised learning and context prediction on molecules from ChEMBL [44].
- Mol2vec: A variant of the word2vec model trained on 22 million molecules from the ZINC database [45].

Table 2 contains a detailed overview of all benchmarks. Based on our benchmarking, we observed that tasks 2 (Table 4) and 3 (Table 5) were challenging for all methods. As can be seen in Table 3, SMR-DDI performs relatively well when comparing metrics such as F1-Score, Precision, Recall, AUPRC, and AUROC in Task 1. SMR-DDI performs similarly well to Mol2vec and MACCs Keys and outperforms ChemGPT-1 and ChemGPT-4, emphasizing the relevance of the captured features. In addition, SMR-DDI performs similarly to MACCkeys on tasks 2 and 3 and still outperforms ChemGPT-1 and ChemGPT-4. In particular, on task 3, SMR-DDI performs similarly to ChemBerta-77 M-MLM and ChemBerta-77 M-MLR and outperforms ChemGPT-4 and ChemGPT-1. Although SMR-DDI was trained on a smaller dataset (~ 200 K = 2% of the smallest pre-training benchmark dataset), it performs comparably to larger models. This shows that it can learn effectively from a limited amount of data with data augmentation. However, it performed slightly worse than Mol2vec on tasks 2 and 3, suggesting that the learned features may be too invariant or insufficiently rich. The performance of SMR-DDI is also slightly lower than some other methods, such as gin_supervised_infomax, gin_supervised_contextpred, and gin_supervised_edgepred, which are graph-based models trained on larger datasets (ChEMBL + ZINC15 with 465,000 + 2 million training molecules). This suggests that the larger and more diverse training datasets may contribute to their superior performance. A larger training dataset can provide more diverse and representative samples, potentially improving the performance of the model (Tables 4 and 5).

**Table 2:** Description of all molecular featurizers benchmarks

Featurizer	Type	Dataset name	Dataset size	Feature vector. dim	Architecture
ECFP	Hashed fingerprint	–	–	2048	–
ChemBERTa-77M	Pretrained	PubChem	77 M	384	Transformer
MOL2VEC	Pretrained	ZINC + ChemBL	19.9 M	300	Word2vec
SMR-DDI	Pretrained	Chembl	200 K	262	CNN
ChemGPT-1B	Pretrained	PubChem	10 M	256	Transformer
MACCKEYS	Structural fingerprint	–	–	166	–
gin_supervised_edgepred	Pretrained	ChembL + ZINC15	465 K + 2 M	300	Graph
ChemGPT-4M	Pretrained	PubChem	10 M	128	Transformer
gin_supervised_contextpred	Pretrained	ChembL + ZINC15	465 K + 2 M	300	Graph
gin_supervised_masking	Pretrained	ChembL + ZINC15	465 K + 2 M	300	Graph
gin_supervised_masking	Pretrained	ChembL + ZINC15	465 K + 2 M	300	Graph

**Table 3:** Performance of SMR-DDI and other featurizers on Task 1

Featurizer	AUPRC	AUROC	ACC	F1	Precision	Recall	
ECFP	0.942 ± 0.02	0.996 ± 0.002	0.954 ± 0.002	0.954 ± 0.002	0.954 ± 0.002	0.954 ± 0.002	
gin_supervised_infomax	0.91 ± 0.013	0.994 ± 0.002	0.93 ± 0.003	0.93 ± 0.003	0.93 ± 0.003	0.93 ± 0.003	
gin_supervised_contextpred	0.902 ± 0.02	0.993 ± 0.003	0.928 ± 0.003	0.928 ± 0.003		0.928 ± 0.003	0.928 ± 0.003
ChemBERTa-77 M-MLM	0.918 ± 0.008	0.993 ± 0.004	0.924 ± 0.006	0.924 ± 0.006	0.925 ± 0.005	0.924 ± 0.006	
gin_supervised_masking	0.9 ± 0.023	0.993 ± 0.003	0.923 ± 0.006	0.923 ± 0.006		0.923 ± 0.005	0.923 ± 0.006
gin_supervised_edgepred	0.918 ± 0.013	0.992 ± 0.003	0.923 ± 0.003	0.923 ± 0.003	0.924 ± 0.003	0.923 ± 0.003	
ChemBERTa-77 M-MLR	0.917 ± 0.016	0.992 ± 0.004	0.907 ± 0.007	0.908 ± 0.007	0.908 ± 0.007	0.908 ± 0.007	
MACCKEYS	0.919 ± 0.016	0.994 ± 0.003	0.892 ± 0.022	0.893 ± 0.022	0.895 ± 0.02	0.892 ± 0.022	
SMR-DDI	**0.9 ± 0.005**	**0.992 ± 0.003**	**0.877 ± 0.014**	0.877 ± 0.014	**0.88 ± 0.013**	**0.877 ± 0.014**	
MOL2VEC	0.91 ± 0.006	0.992 ± 0.002	0.869 ± 0.028	0.869 ± 0.027	0.873 ± 0.023	0.869 ± 0.028	
ChemGPT-4	0.875 ± 0.017	0.993 ± 0.002	0.847 ± 0.026	0.848 ± 0.026	0.854 ± 0.022	0.847 ± 0.026	
ChemGPT-1	0.877 ± 0.017	0.99 ± 0.004	0.839 ± 0.047	0.839 ± 0.047	0.846 ± 0.041	0.839 ± 0.047	

SMR-DDI results are in bold

**Table 4:** Performance of SMR-DDI and other featurizers on Task 2

Featurizer	AUPRC	AUROC	ACC	F1	Precision	Recall
gin_supervised_contextpred	0.503 ± 0.039	0.908 ± 0.023	0.596 ± 0.005	0.583 ± 0.007	0.599 ± 0.01	0.596 ± 0.005
ECFP	0.502 ± 0.047	0.897 ± 0.036	0.601 ± 0.016	0.581 ± 0.022	0.616 ± 0.017	0.601 ± 0.016
gin_supervised_masking	0.494 ± 0.051	0.901 ± 0.019	0.594 ± 0.008	0.579 ± 0.009	0.602 ± 0.006	0.594 ± 0.008
gin_supervised_infomax	0.472 ± 0.065	0.884 ± 0.031	0.589 ± 0.004	0.576 ± 0.007	0.593 ± 0.01	0.589 ± 0.004
gin_supervised_edgepred	0.48 ± 0.036	0.905 ± 0.024	0.585 ± 0.01	0.57 ± 0.012	0.593 ± 0.013	0.585 ± 0.013
MOL2VEC	0.554 ± 0.022	0.913 ± 0.006	0.575 ± 0.024	0.565 ± 0.022	0.573 ± 0.023	0.575 ± 0.024
ChemBERTa-77M-MLM	0.491 ± 0.021	0.9 ± 0.02	0.58 ± 0.013	0.564 ± 0.017	0.585 ± 0.013	0.58 ± 0.013
ChemBERTa-77M-MLR	0.533 ± 0.016	0.911 ± 0.009	0.571 ± 0.014	0.562 ± 0.01	0.575 ± 0.013	0.571 ± 0.014
MACCKEYS	0.482 ± 0.028	0.891 ± 0.014	0.543 ± 0.015	0.528 ± 0.014	0.545 ± 0.014	0.543 ± 0.015
SMR-DDI	0.434 ± 0.025	0.896 ± 0.008	0.528 ± 0.005	0.51 ± 0.006	0.529 ± 0.007	0.528 ± 0.005
ChemGPT-4	0.447 ± 0.035	0.899 ± 0.01	0.503 ± 0.014	0.486 ± 0.018	0.499 ± 0.021	0.503 ± 0.014
ChemGPT-1	0.452 ± 0.039	0.898 ± 0.015	0.493 ± 0.01	0.476 ± 0.011	0.488 ± 0.011	0.493 ± 0.01

**Table 5:** Performance of SMR-DDI and other featurizers on Task 3

Featurizer	AUPRC	AUROC	ACC	F1	Precision	Recall
MOL2VEC	0.296 ± 0.035	0.786 ± 0.031	0.343 ± 0.019	0.338 ± 0.017	0.352 ± 0.019	0.343 ± 0.019
gin_supervised_edgepred	0.233 ± 0.053	0.744 ± 0.029	0.345 ± 0.015	0.327 ± 0.015	0.343 ± 0.017	0.345 ± 0.015
ECFP	0.251 ± 0.023	0.767 ± 0.04	0.34 ± 0.014	0.323 ± 0.018	0.343 ± 0.01	0.34 ± 0.014
MACCKEYS	0.227 ± 0.07	0.71 ± 0.058	0.338 ± 0.023	0.317 ± 0.022	0.336 ± 0.019	0.338 ± 0.023
gin_supervised_infomax	0.228 ± 0.035	0.704 ± 0.038	0.339 ± 0.017	0.315 ± 0.019	0.339 ± 0.015	0.339 ± 0.017
gin_supervised_contextpred	0.244 ± 0.029	0.752 ± 0.028	0.344 ± 0.022	0.313 ± 0.016	0.336 ± 0.017	0.344 ± 0.022
gin_supervised_masking	0.225 ± 0.034	0.723 ± 0.03	0.328 ± 0.007	0.311 ± 0.008	0.339 ± 0.018	0.328 ± 0.007
ChemBERTa-77 M-MLM	0.239 ± 0.042	0.734 ± 0.029	0.334 ± 0.028	0.311 ± 0.026	0.336 ± 0.029	0.334 ± 0.028
ChemBERTa-77 M-MLR	0.249 ± 0.025	0.755 ± 0.027	0.318 ± 0.018	0.302 ± 0.016	0.319 ± 0.022	0.318 ± 0.018
SMR-DDI	0.154 ± 0.019	0.739 ± 0.041	0.305 ± 0.014	0.295 ± 0.013	0.312 ± 0.014	0.305 ± 0.014
ChemGPT-1.2B	0.165 ± 0.016	0.739 ± 0.035	0.279 ± 0.014	0.263 ± 0.018	0.271 ± 0.022	0.279 ± 0.014
ChemGPT-4 M	0.163 ± 0.019	0.749 ± 0.026	0.262 ± 0.021	0.254 ± 0.016	0.274 ± 0.015	0.262 ± 0.021

Although graph methods seem appealing, they are not explicitly designed for SMILES enumeration, as the molecular graph remains unchanged for canonical and randomized SMILES. The advantage of SMILES enumeration lies in its ability to generate alternative readings by generating variable permutations of the molecular graph. One idea to explore is the development of a propagation algorithm that updates the nodes of the molecular graph based on a specific permutation order.

Another factor that could influence the quality of the vector representation, besides the choice of training dataset, is the size of the feature space. SMR-DDI has a feature vector dimension of 262, which is relatively low compared to some other models, such as ECFP, MOL2VEC, and ChemBERTa-77 M, which have higher dimensional feature vectors (e.g., 2048, 300, and 384, respectively). Higher dimensional feature vectors can capture more detailed and informative representations of drugs, improving the model's ability to learn complex relationships and patterns. When working with small training datasets, as we do, the primary concern is overfitting. Overfitting occurs when a model becomes too specialized in learning from the limited data it has seen during training, resulting in poor generalization to unseen data. A higher embedding space or higher dimensional embeddings could potentially increase the risk of overfitting, especially with limited training data [46, 47]. It will be worth exploring a higher embedding space with more training data.

Overall, the performance of SMR-DDI is encouraging and suggests its effectiveness in predicting side effects between known drug pairs. However, for a comprehensive evaluation and comparison, it is essential to consider factors such as the size and diversity of the training dataset and the dimension of the feature vector space.

### Ablation study on pre-training

We conducted an ablation study to evaluate the effects of the pretraining phase. We applied our fully connected classifier to the initial feature space and retrained the model using three evaluation schemes. As depicted in Table 6, using the pre-trained feature space leads to superior performance compared to using the initial feature space. The pre-trained SMR-DDI model showed a significant increase in performance compared to the non-pre-trained SMR-DDI model, with an average increase in F1 score of 6.5% on the tree tasks, for example. This result emphasizes the importance of the pre-training phase for improving the model's ability to learn discriminative features. Using the knowledge acquired during pre-training on ChEMBL, the model can extract more informative representations, leading to better results in all three evaluation tasks. This experiment confirms our initial hypotheses (2b and 3) about how relevant pre-training improves the performance of the prediction model.

**Table 6:** Ablation study results

Model	AUPRC	AUROC	ACC	F1	Precision	Recall
*Task 1*						
SMR-DDI_*unpretrained*_	0.788 ± 0.04	0.986 ± 0.006	0.713 ± 0.063	0.712 ± 0.065	0.743 ± 0.046	0.713 ± 0.063
SMR-DDI	0.9 ± 0.005	0.992 ± 0.003	0.877 ± 0.014	0.877 ± 0.014	0.88 ± 0.013	0.877 ± 0.014
*Task 2*						
SMR-DDI_*unpretrained*_	0.315 ± 0.011	0.878 ± 0.01	0.419 ± 0.027	0.414 ± 0.022	0.436 ± 0.022	0.419 ± 0.027
SMR-DDI	0.434 ± 0.025	0.896 ± 0.008	0.528 ± 0.005	0.51 ± 0.006	0.529 ± 0.007	0.528 ± 0.005
*Task 3*						
SMR-DD_I*unpretrained*_	0.089 ± 0.018	0.687 ± 0.014	0.161 ± 0.047	0.168 ± 0.029	0.208 ± 0.003	0.161 ± 0.047
SCL-DDI	0.154 ± 0.019	0.739 ± 0.041	0.305 ± 0.014	0.295 ± 0.013	0.312 ± 0.014	0.305 ± 0.014

### Influence of the dataset molecular diversity

**Fig. 12:**
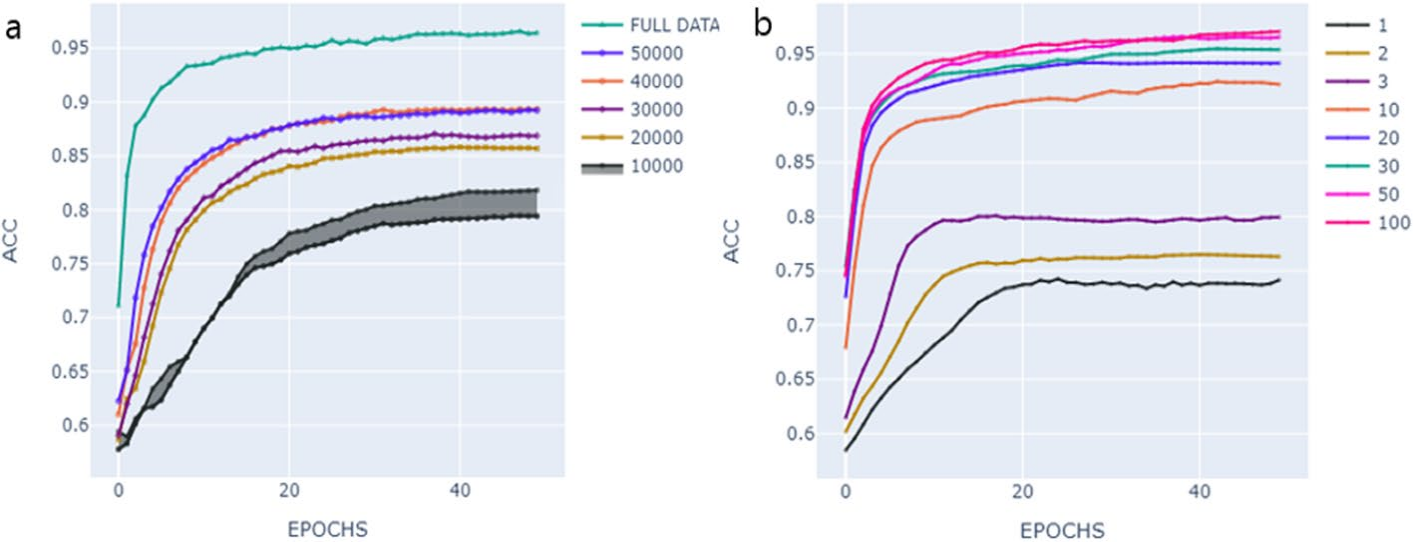
Influence of Molecular Diversity on Training Performance. **a** Influence of Scaffolds Diversity on Training Performance. **b** Impact of Number of SMILES per Scaffold on Training Performance

In this section, we aim to investigate the influence of molecular diversity in the pre-training dataset on the quality of the learned representation. We investigated how the model performs when we add more scaffolds during training. We randomly selected five batches with 10,000 different scaffold families each. The model was trained with each set of 10,000 families separately. We then created incremental batches, starting with a batch size of 20,000 families and gradually increasing to 30,000, 40,000, and 50,000. We repeated this iterative process several times and reported the results and standard deviation (Fig. 12a). For illustrative purposes, only the standard deviation for groups of 10,000 is shown in the figure, as the observed trend is consistent even for larger training sets.

To compare the different experiments, we utilized the “acc_top1” metric. This metric quantifies the frequency at which the randomized view (correct view) ranked within the top-1 most similar views in the batch when considering the learned representation for Drugbank drugs. We noticed that the model’s ability to discriminate improves when we include more families. The increase in performance is also proportional to the structural diversity introduced by each new set of families, as shown by the Similarity to the nearest neighbor (SNN) and Scaffold similarity (Scaff) metrics reported in Table 7. SNN and Scaff [48] metrics are used to assess how similar scaffolds are in different molecular datasets. Higher values of SNN and Scaff indicate low structural variability in the evaluated datasets, suggesting that the datasets contain molecules with similar structural scaffolds. These observations emphasize the ability of the SMR-DDI molecular representation to adapt and evolve with increasing diversity in the dataset.

**Table 7:** Similarity to the nearest neighbor (SNN) and Scaffold similarity (Scaff) metrics

Datasets	SNN ($\uparrow$)	Scaff ($\uparrow$)
10,000–20000	0.7697	0.9220
20,000–30000	0.8696	0.9692
30,000–40000	0.9136	0.9845
40,000–50000	0.9370	0.9902

In the subsequent phase, we selected a fixed number of families of molecules and gradually increased the number of representatives (drugs) per family by the 1 ×, 2 ×, 3 ×, 10 ×, 20 ×, 30 ×, 50 ×, and 100 × for each batch. Throughout the process, we monitored the top-1 accuracy (top1-acc) as we incrementally increased the number of drugs per scaffold. Figure 12b shows the relationship between the number of SMILES (molecules) per scaffold and training performance. Increasing the number of SMILES per scaffold enables faster convergence (fewer epochs) of the learning curve. However, increasing the number of drugs per scaffold above a certain threshold does not improve the learning of meaningful representations and does not hinder effective training. We have indeed observed that the performance gains become less significant after reaching a 50-fold increase. This observation could be due to the ability of SMR-DDI to generate new examples from existing SMILES and confirm that relevant data augmentation through SMILES enumeration increases the diversity of the data (Hypothesis 1b).

These results indicate that the molecular diversity within the pre-training dataset plays a crucial role in the quality of the learned representation. Higher diversity facilitates more effective learning of the model and accelerates the achievement of peak performance.

## Conclusion

To summarize, we have developed and evaluated SMR-DDI. This self-supervised framework uses contrastive learning to embed drugs into a scaffold-based feature space to predict drug–drug interactions (DDI). The framework was pre-trained on a large unlabeled molecule dataset and used SMILES enumeration to generate augmented views for each molecule. The pre-trained model demonstrated its ability to learn abstract, transferable features from a large unlabeled molecular dataset. The learned representations were shown to be expressive, yielding comparable or better results for DDI prediction compared to state-of-the-art molecular representations. Furthermore, our investigation of interaction and side-effect profiles improved our understanding of the characteristics and behavior of molecules within the dataset. We have identified patterns and associations between specific scaffold types, their corresponding interactions, and side effect profiles, allowing for a more nuanced assessment of drug interactions. Our results highlighted the potential of contrastive learning as a promising approach for DDI prediction and emphasized the importance of expressive feature space for accurately classifying molecules and capturing their subtleties.

### Supplementary Information

**Additional file 1**. Distribution of Scaffold Combinations.

**Additional file 2**. FP-GRowth : Most frequents itemsets.

**Additional file 3**. Apriori rules on Drugbank.

**Additional file 4**. Scaffold clustering based on their interactions profiles.

**Additional file 5**. Most challenging side effects to predict.

**Additional file 6**. Pairs of side effects characterized by the same sets of scaffolds where they occur.

**Additional file 7**. LDA visualization of molecular representations learned by SMR-DDI for highly similar molecules.

**Additional file 8**. Data imbalance : Impact of different sampling strategies on DDI prediction performances.

## Data Availability

The code is available at https://github.com/srkpa/SMRDDI.

## Footnotes

1 https://tdcommons.ai/multi_pred_tasks/ddi/

2 https://github.com/deepchem/deepchem#citing-deepchem
