# Supplementary figures and images for: Learning self-supervised molecular representations for drug–drug interaction prediction
Source: BMC Bioinformatics. 2024 Jan 30;25:47. doi: 10.1186/s12859-024-05643-7 (PMC10829170; doi:10.1186/s12859-024-05643-7)

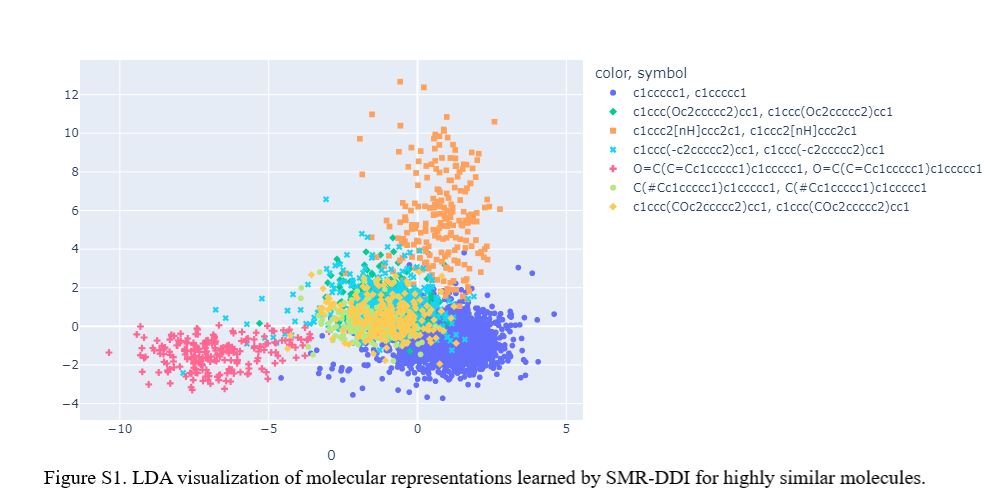

Supplement: Supplementary file 7 — Additional file 7. LDA visualization of molecular representations learned by SMR-DDI for highly similar molecules. [file 12859_2024_5643_MOESM7_ESM.png]

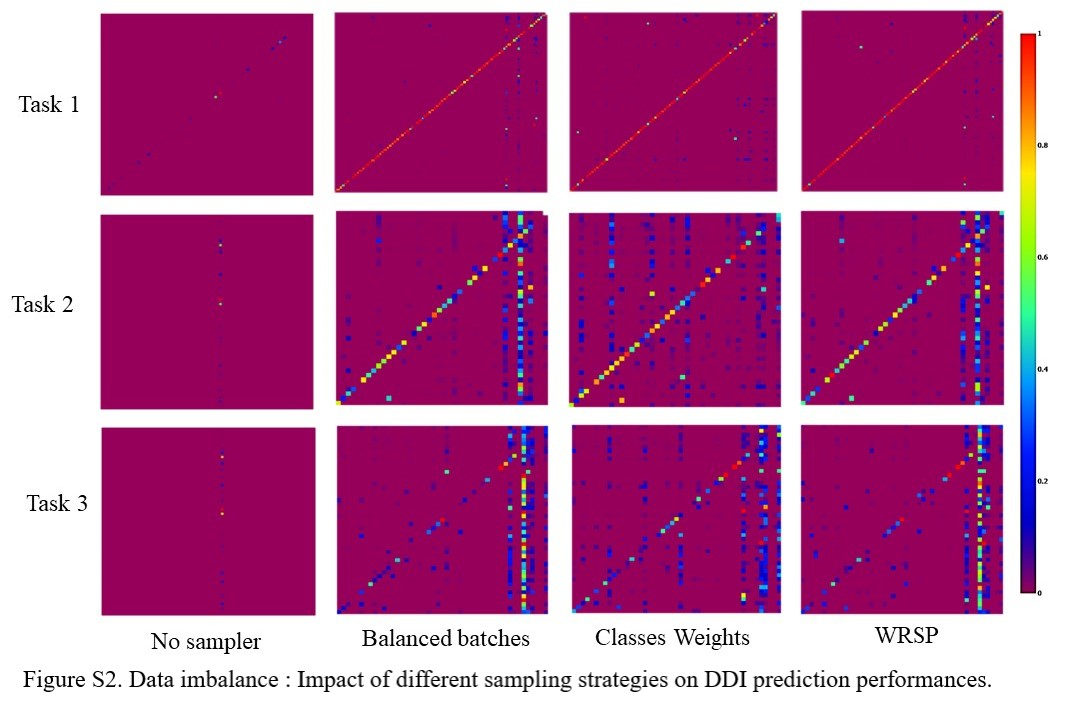

Supplement: Supplementary file 8 — Additional file 8. Data imbalance : Impact of different sampling strategies on DDI prediction performances. [file 12859_2024_5643_MOESM8_ESM.png]
